# Cross-Sectional Imaging of the WS_2_ Nanotube Formation Pathways

**DOI:** 10.1021/acs.nanolett.6c00855

**Published:** 2026-04-08

**Authors:** Kristyna Bukvisova, Libor Novak, Vojtech Kundrat

**Affiliations:** † Thermo Fisher Scientific, Vlastimila Pecha 12, 62700 Brno, Czech Republic; ‡ Department of Chemistry, 117204 Faculty of Science, Masaryk University, Kamenice 5, Brno 62500, Czech Republic

**Keywords:** WS_2_ nanotube, sulfidation, cross-section, lamella, electron microscopy, reaction mechanism

## Abstract

Recent elucidation of the synthesis mechanism of WS_2_ nanotubes has raised fundamental questions about the origin of structural subtypes, imperfect nanotubes, and internal scroll-like morphologies. Although such 1D structures are observed in both laboratory and industrial batches, their formation pathways remain unclear. As applications of WS_2_ nanotubes—particularly in optoelectronics—continue to expand, understanding these structural variations is essential for process optimization and performance control. Here, we develop an advanced imaging approach that combines stepwise sulfidation on a microelectromechanical-system chip with sequential cross-sectional imaging of individual nanostructures. This methodology enables time-resolved, atomic-scale correlation between the internal structure of tungsten oxide nanowhisker precursors and the resulting WS_2_ morphologies. We identify multiple reaction pathways and establish direct links between precursor anisotropy and nanotube geometry, refining the sulfidation mechanism and clarifying the transformation from tungsten oxide to tungsten disulfide. These insights provide a framework for controlled synthesis of structurally optimized WS_2_ nanotubes.

## Graphical abstract

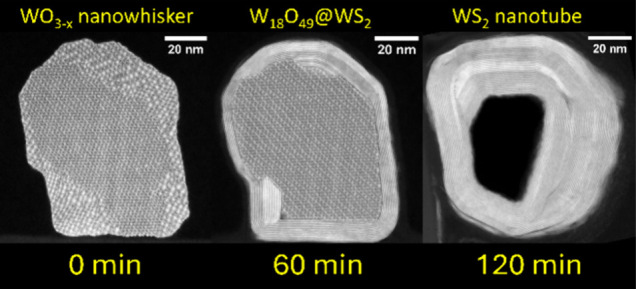

Precision in nanomaterial synthesis is essential for their integration into advanced technologies. Structural perfection—ideally a defect-free architecture—ensures uniform optoelectronic, mechanical, and chemical properties and facilitates translation from laboratory research to practical nanoengineered devices. Assessing and improving this structural precision is therefore central to the development of any nanomaterial system. Here, we focus on WS_2_ nanotubes,^1–5^ quasi-one-dimensional inorganic nanostructures that initiated the field of inorganic nanotube research and already offer a multitude of advanced applications ranging from optoelectronics^3,6–10^ to reservoir computing^11^ or the immobilization of actinides.^12^ These materials can be viewed as curved transition-metal dichalcogenide layers that overcome elastic bending constraints and close into cylinders. They are typically synthesized via high-temperature sulfidation of tungsten suboxide nanowhiskers (e.g., W_18_O_49_) in an H_2_S/H_2_ or sulfur vapor atmosphere through a self-sacrificial template mechanism.^2,13–16^ Alternatively, high-quality nanotubes with nearly perfect cylindrical profiles were grown in a one-step process from tungsten oxide nanoparticles.^17,18^

Using combined in situ and ex-situ electron microscopy, we recently elucidated the reaction pathway,^19^ which proceeds through two consecutive routes. In the initial *surface-inward* mechanism, sulfur species react with tungsten oxide to form a passivating WS_2_ shell. This is followed by the *receding-oxide-core* mechanism, in which the oxide core evaporates anisotropically from the nanowhisker tips, forming an internal cavity. Concurrently, additional WS_2_ layers nucleate internally via gas-phase reactions between evaporated tungsten oxide species and the H_2_S/H_2_ atmosphere until the precursor is fully consumed.

Although optimization yields thin, symmetric nanotubes (Figure 1a) with circular cross sections^14,15,17,17^ (Figure 1b), multiwalled nanotubes with significant variation in diameter and internal architecture are common, especially if a two-step reaction protocol is used with separate growth of tungsten suboxide nanowhiskers followed by high-temperature sulfidation. Defective structures—particularly thicker nanotubes exhibiting asymmetry, multiple cavities, bundled morphologies, or internally folded layers—are consistently present (Figure S1a–c).^20,21^ In cross-section, some nanotubes contain randomly folded WS_2_ layers and multiple voids (Figure 1c), while side-view imaging often suggests nearly filled interiors with only small irregular cavities (Figure S1b). Despite being structurally identifiable as WS_2_ nanotubes, imperfections such as wall-thickness asymmetry, misoriented layers, and internal irregularities remain prevalent, and their origin is unresolved. A detailed understanding of how the atomic structure of the tungsten suboxide precursor governs sulfidation and the final nanotube morphology is still lacking. Atomically resolved insight would enable rational control of the transformation pathway and potentially scalable structural optimization. Improved structural control would enhance optical, electronic, and mechanical performance, enabling applications ranging from ultralong filtration meshes to strain-engineered core–shell heteronanotubes with programmable optoelectronic functionality. Importantly, full control over the oxide-chalcogenide interface could be highly beneficial for obtaining precisely engineered heterogeneous structures such as WS_2_@MoS_2_,^22^ MoO_3_@MoS_2_,^23^ or WTe_2_@WS_2_.^24^

**Figure 1:**
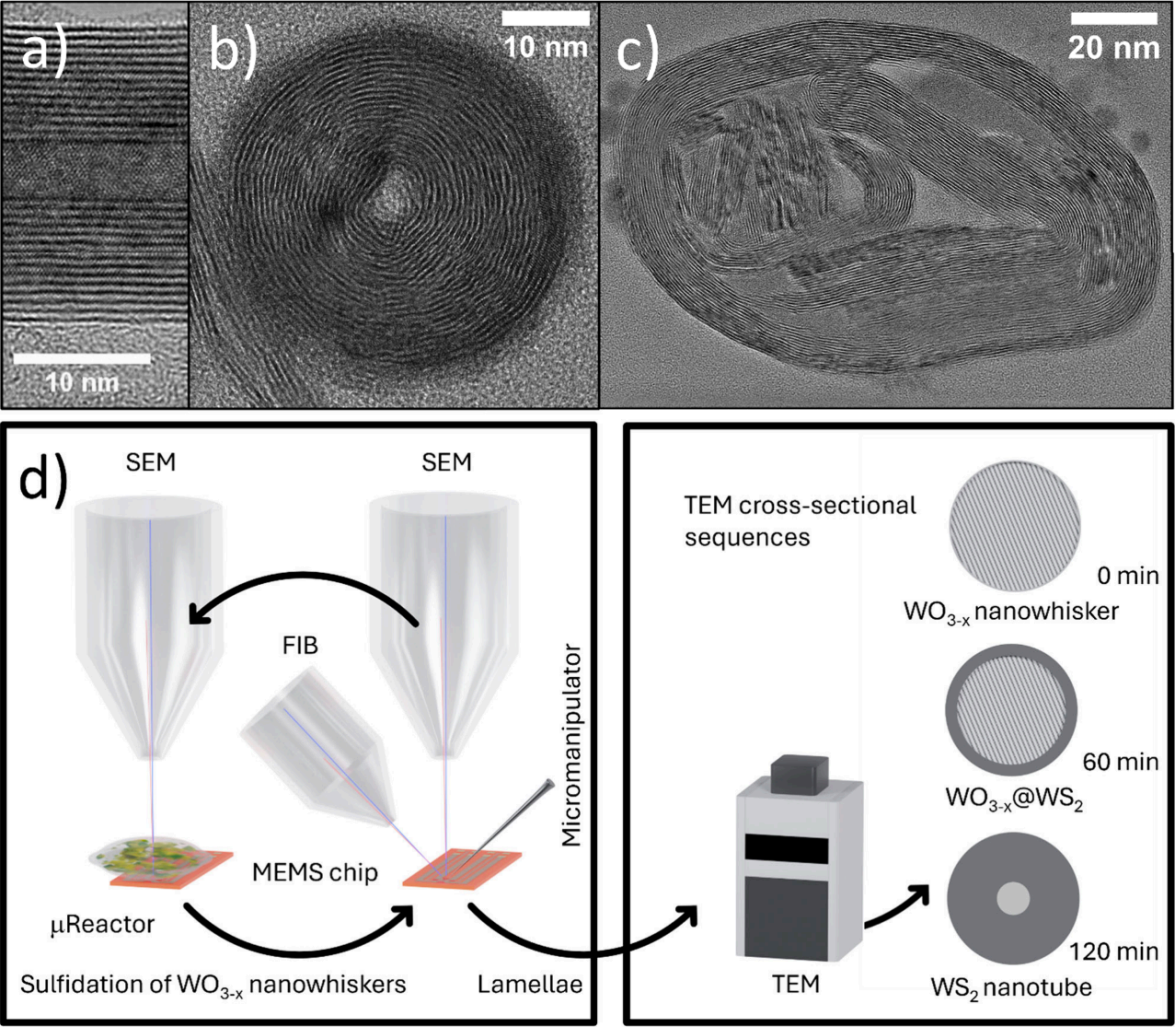
TEM analyses of WS_2_ nanotubes with a perfect cylindrical shape, viewed from the side (a) and as a cross-section (b). Cross-section of WS_2_ nanotube with internal architecture shaped into scrolls, plates, and multiple cavities (c). Graphical scheme of the developed methodology (d). A single, elongated W_18_O_49_ nanowhisker is first preselected on a MEMS chip. An initial lamella is lifted out using FIB and a micromanipulator to obtain the 0 min reference state (prior to sulfidation). The MEMS chip carrying the remaining nanowhisker is then subjected to controlled sulfidation in an H_2_S/H_2_ atmosphere inside the μ-reactor integrated within the SEM chamber. After a defined reaction time, a second lamella is extracted from the same nanowhisker. The sulfidation–lift-out sequence is repeated multiple times, generating a time-resolved series of cross-sectional specimens. These sequential lamellae are subsequently analyzed by TEM, enabling the reconstruction of the reaction progression from nanowhisker to nanotube.

Direct correlation between precursor structure and transformation dynamics requires tracking individual W_18_O_49_ nanowhiskers during sulfidation. Recent advances in scanning electron microscopy enable in situ high-temperature reactions using a μReactor^19,25,26^ platform operating at low pressure (up to 500 Pa) with microelectro-mechanical-system (MEMS) chips^27,28^ that serve simultaneously as heaters, sample holders, and transmission electron microscopy support grids.^26^ Rapid heating and cooling enable controlled interruption of the reaction, allowing continuous in situ SEM observation of morphology and kinetics, followed by ex-situ atomic-scale TEM analysis of phase transformations, mass transport, and structural rearrangements.

Previous studies monitored sulfidation primarily from a side view, clarifying the global mechanism but not the influence of precursor internal morphology.^19^ Here, we extend the in situ/ex-situ SEM–TEM approach to perform stepwise cross-sectional analysis along the ⟨010⟩ axis of individual W_18_O_49_ ultralong nanowhiskers. An initial lamella is extracted from the pristine nanowhisker; the remaining structure undergoes controlled sulfidation, interrupted at predefined times to extract additional lamellae from distinct regions of the evolving nanotube. This methodology produces time-resolved cross-sectional sequences from a single transforming nanostructure. The developed approach is depicted as pictographic Figure 1d. Our goal is to establish a direct atomistic correlation between the precursor structure and the nanotube formation pathway, thereby elucidating the origins of structural irregularities, defect formation, and morphological diversity in WS_2_ nanotubes. The results and mechanistic interpretation of this investigation follow.

As the precursor for nanotube synthesis and subsequent cross-sectional analysis, ultralong W_18_O_49_ nanowhiskers were prepared using a modified version of a previously reported protocol.^29^ A series of controlled sulfidation experiments was performed to monitor nanotube formation. From these specimens, a single nanowhisker approximately 80 nm in thickness, tens of micrometers in length, and exhibiting a slightly oval cross-sectional profile was selected for detailed investigation (SEM image prior to lamella extraction shown in Figure 2a). During stepwise sulfidation, six lamellae were sequentially lifted out at predefined reaction times (0 min—pristine state, 3, 60, and 120 min—3 different lamellae) and subsequently analyzed by high-resolution scanning transmission electron microscopy in high-angle annular dark-field mode (HRSTEM–HAADF).

**Figure 2:**
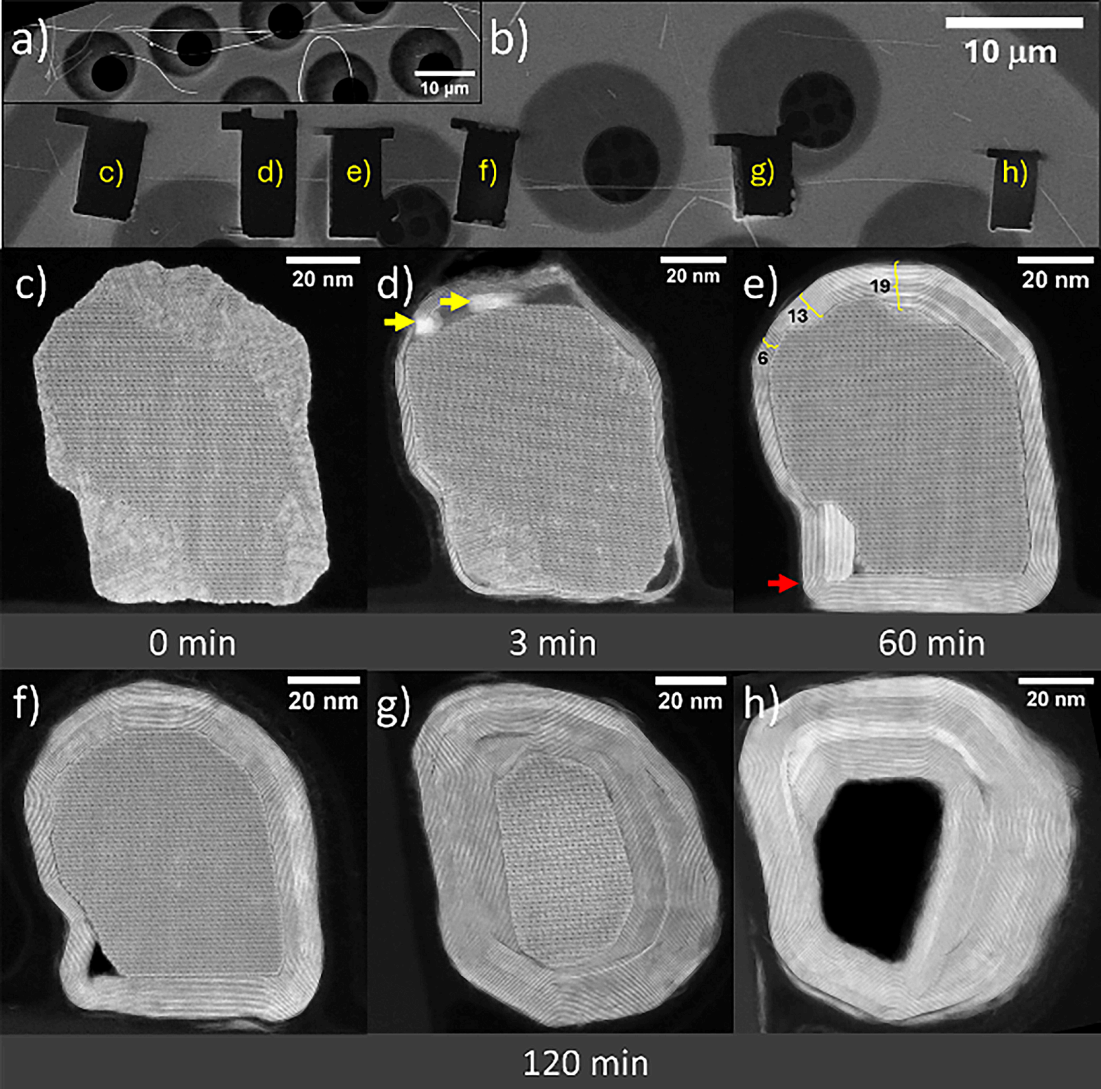
Cross-sectional imaging sequence of the sulfidation of W_18_O_49_ nanowhisker along the ⟨010⟩ direction and nanotube axis. (a) SEM image of the tungsten oxide nanowhisker selected for the cross-sectional sequential analysis. (b) SEM image of the same nanowhisker after the sulfidation reactions and lamellae lifting up. (c) The TEM image at 0 min shows the inner structure before sulfidation. Further sulfidation (d) and (e) shows the ingrowth of WS_2_ layers toward the tungsten oxide core. (f–h) 3 cross sections were lifted out at different places along the nanotube, showing various degrees of sulfidation reaction. From a moderate state in (f) where the tungsten oxide is still the predominant phase, across the intermediate state (g) where tungsten oxide is nearly consumed, until (h) where the hollow WS_2_ nanotube is displayed as a cross-section.

The SEM image of the specimen after extraction of all six lamellae is shown in Figure 2b. The cross-section of the pristine nanowhisker (0 min) is presented in Figure 2c. Although initially appearing oval in SEM, the nanowhisker cross-section is in fact polygonal and composed of two distinct structural segments. The central region consists of single-crystalline W_18_O_49_, surrounded by a ring of higher tungsten oxide containing structural motifs of the W_20_O_58_ phase. A higher-magnification image corresponding to Figure 2c is provided in the Supporting Information (Figure S2).

After 3 min of sulfidation, a thin shell composed of 2–5 WS_2_ layers formed around the nanowhisker (Figure 2d). Notably, cavities developed near the polygonal corners of the nascent nanotube. This observation indicates that oxide evaporation occurs not only from the nanowhisker tips but also laterally from within the forming nanotube. This behavior agrees with previously reported anisotropic evaporation observed by in situ SEM and ex-situ TEM.^19^ Tungsten suboxide evaporates preferentially along ⟨010⟩ as individual shear planes or entire domains.^26^ Bright contrast features (yellow arrows) partially filling the voids correspond to an amorphous tungsten-rich phase precipitated during rapid cooling. A higher-resolution image of Figure 2d is shown in Figure S3.

Sulfidation was continued and interrupted again after 60 min (Figure 2e). At this stage, the W_20_O_58_ phase was completely consumed and converted into WS_2_, while the remaining oxide core consisted solely of W_18_O_49_. The overall shape of the original nanowhisker was largely preserved, although the upper region became more rounded. In contrast, the bottom part remained nearly flat and aligned with the hexagonal channels of the W_18_O_49_ lattice (see high-resolution image in Figure S4). The WS_2_ wall thickness varies across the cross-section, indicating nonuniform inward growth (yellow brackets in Figure 2e). In the lower region, WS_2_ layers exhibit a 90° bend (red arrow), suggesting that substantial strain develops during the oxide-to-sulfide transformation at elevated temperature. The direct effect of strain within the structure can be observed in changes in the interlayer distance. While the usual spacing between WS_2_ layers in the cylindrical nanotube is 0.63–0.65 nm,^30^ the Fast Fourier Transformation (FFT) analysis showed a local deviation of 0.61 nm distance between layers that form a 90° angle (bottom part of Figure S4).

After 120 min of total reaction time, three additional cross sections were extracted from different locations along the same nanotube (Figure 2f–h). Two regions still contained a residual oxide core, whereas one region was already fully hollow. Thus, complete conversion was not achieved uniformly along the structure within 120 min. Comparison of Figures 2f and 2g highlights significant variation: in Figure 2f, a tungsten oxide core is enclosed by approximately 20 WS_2_ layers, whereas in Figure 2g the oxide is nearly fully converted, forming ∼40 WS_2_ layers. In Figure 2h, the nanotube is entirely hollow. These differences demonstrate that sulfidation progresses heterogeneously along the nanowhisker, with locally varying WS_2_ growth rates. All three images are available in high resolution in the Supporting Information as Figures S5–S7.

Importantly, the precursor geometry strongly dictates the resulting nanotube morphology. The nanotube diameter closely matches that of the original nanowhisker, and the overall cylindrical shape reflects the initial structure. However, internally folded WS_2_ layers do not always form a closed cylinder; instead, scroll-like architectures are observed (Figure 2h). This behavior may originate from the heterogeneous phase composition and domain structure of the precursor (Figure 2a).

The heterogeneous reaction progression along the nanotube is further supported by TEM analysis shown in Figure 3. A partially converted nanowhisker (reaction interrupted after 10 min in a conventional flow reactor) is presented in Figure 3a. Figures 3b–d provide higher-magnification views: Figure 3b shows the tip of the tungsten oxide core, while Figures 3c and 3d reveal the core–shell structure further along the nanowhisker toward its opposite end. Careful examination of the presented images reveals a fundamentally crucial detail of the so-called *surface-inward* reaction mechanism. The growth of the new WS_2_ layer proceeds along the ⟨010⟩ axis of the W_18_O_49_ lattice from the edges of the tungsten oxide core tip, creating an oxide-sulfide interface, and not laterally from the surface of the nascent nanotube to its core. This could be clearly observed in the detailed Figure 3e-g. Based on this observation, the initial sulfidation step of the reaction mechanism should be refined. At the very first moments of the reaction, the *surface-inward* reaction mechanism creates one to three WS_2_ layers forming the passivation shell, protecting the tungsten oxide core from further sulfidation. Additional layers are formed in the direction and place as suggested by the yellow arrow in Figure 3f. Therefore, the anisotropic character of the tungsten oxide nanowhisker directs both conjoint mechanisms, *surface-inward* and *receding oxide* pathways. Various other supporting examples of WS_2_ nanotubes at different stages of synthesis are shown in Figure S8 of the Supporting Information.

**Figure 3:**
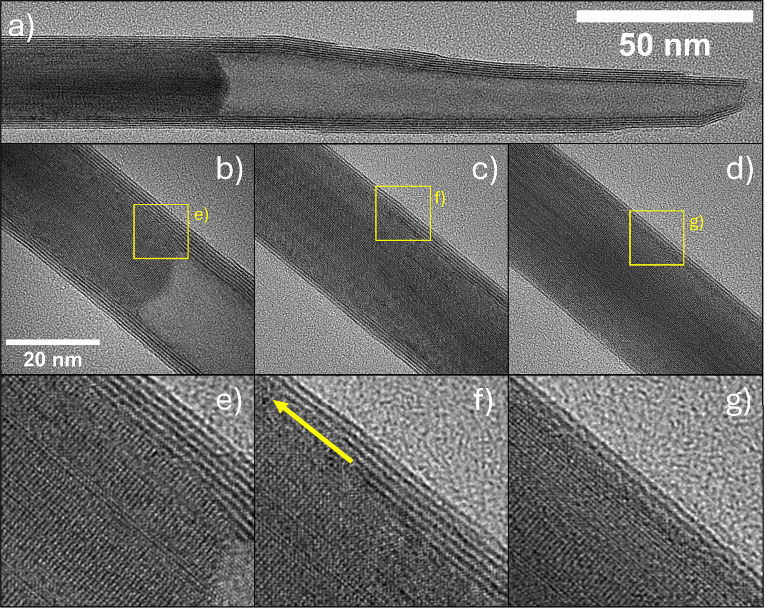
(a) TEM imaging of the WO_3‑x_ nanowhisker sheathed by several layers of WS_2_. (b–d) Selected sections of the nanowhisker along its profile, where (b) displays the tip of the tungsten oxide core and (c and d) different thicknesses of WS_2_ walls further toward the other end of the nanowhisker. (e–g) Corresponding detailed images marked in (b–d).

The structure of tungsten oxide nanowhisker precursors is often multidomain, especially in thicker specimens.^26^ Sulfidation of such a nanowhisker was intentionally followed in Figure 4 as a cross-sectional sequence. The inner structure along the <010> axis displayed at 0 min (before sulfidation) reveals the complex multidomain structure and irregular shape of the nanowhisker. After 3 min of sulfidation reaction, the surface of the whisker was sheathed by two or three layers of WS_2_ with various defects in their structure. The third lamella was lifted out after 2 h of the sulfidation reaction and showed a completely irregular plate-like structure, entirely divergent from the nanotubular cylindrical shape. There are several important observations and deductions based on this sequence. First, the irregular, rough shape of the nanowhisker can prevent the formation of a nanotube. If the passivation WS_2_ layer contains many defects, H_2_S can laterally penetrate the structure, forming plates and irregular, noncylindrical morphologies. WS_2_ nanotube formation is preferred for cylindrical, smooth-surface tungsten oxide nanowhiskers. Second, a thinner, single-domain nanowhiskers facilitate the formation of WS_2_ nanotubes with a perfect cylindrical structure. The importance of crystal domains in sulfidation reactions could be further supported by Figure 5.

**Figure 4:**
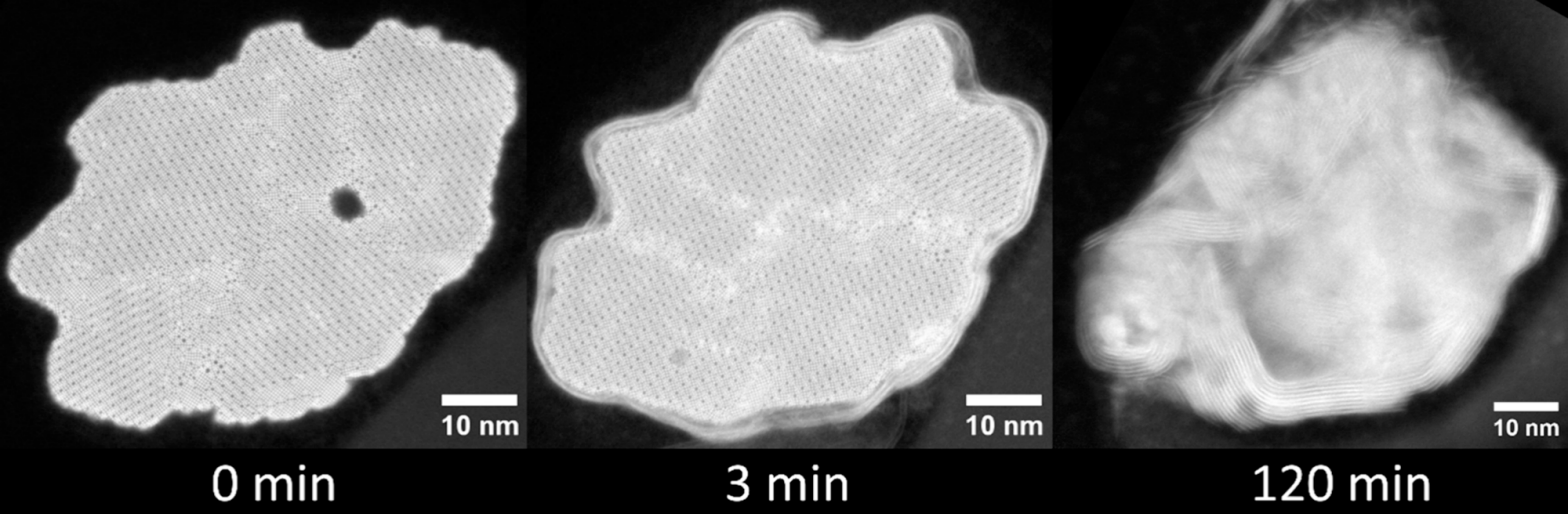
Cross-sectional imaging sequence of the sulfidation of W_18_O_49_ nanowhisker with multiple crystal domains along the ⟨010⟩ direction, resulting in the misaligned WS_2_ 1D-plate structure.

**Figure 5:**
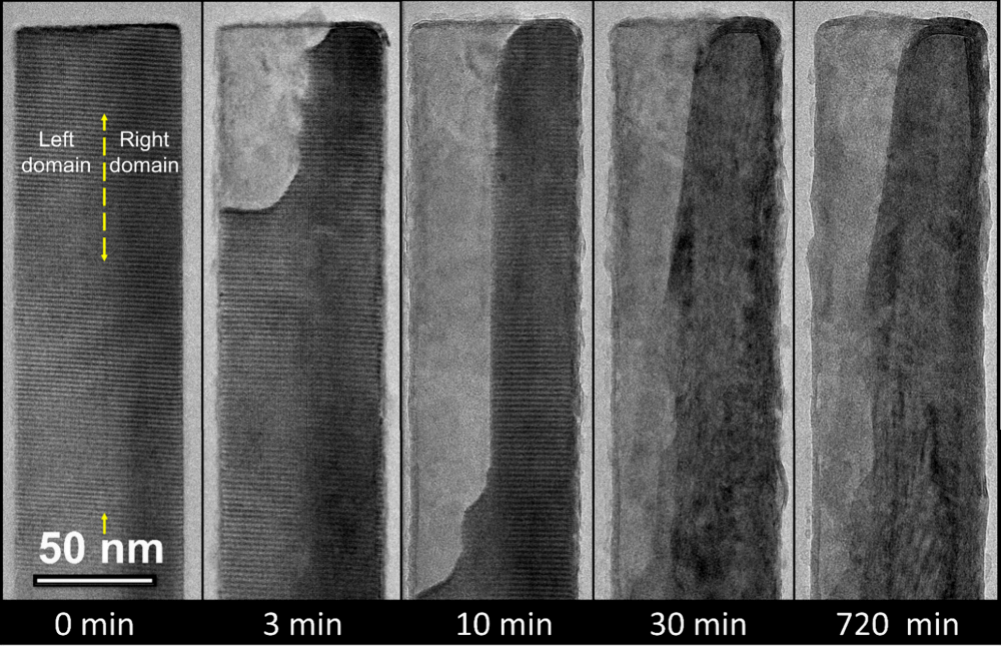
TEM imaging sequence of the sulfidation of tungsten oxide nanowhisker with two domains resulting in the complex misaligned nanotube-like structure. The yellow arrows indicate the domain boundary in pristine nanowhisker.

Here, the sulfidation of the multidomain nanowhisker was followed *ex situ* using a side-view sequential TEM analysis (without lamellae lift out, using methodology described previously^19^). The closer observation of the nanowhisker before sulfidation (0 min) shows an almost indistinct line approximately in the middle of the whisker, indicating a crystalline domain in the structure (marked by a yellow dashed double arrow). After 3 min of sulfidation, the nanowhisker is sheathed by several WS_2_ layers, and the tungsten oxide core begins to evaporate in accordance with the *receding-oxide* mechanism. However, the crystal multidomain character of the nanowhisker is now clearly revealed, as the evaporation rates differ significantly between the left and right domains. After 10 min of sulfidation, the left domain significantly evaporates, and additional layers of the nascent WS_2_ nanotube form, while the right domain remains mostly untouched by the process. Further sulfidation was observed at 30 and 720 min, indicating the formation of an irregular nanotube-like structure within an existing nanotube. Figures S9 and S10 in the Supporting Information show similar pathways for the formation of misaligned nanotubes observed in situ in SEM. Comparatively, the formation of a perfect cylindrical nanotube was previously followed in a similar sequence.^19^

In summary, the time-resolved cross-sectional analysis establishes a direct atomistic correlation between precursor structure and WS_2_ nanotube morphology. Sequential tracking of individual W_18_O_49_ nanowhiskers reveals that sulfidation proceeds anisotropically along the ⟨010⟩ axis, refining the surface-inward mechanism and clarifying how the passivating WS_2_ shell initially forms. The intrinsic crystallographic anisotropy and domain structure of the precursor govern both the receding-oxide pathway and the spatially nonuniform growth of new WS_2_ layers, leading to heterogeneous conversion along a single nanowhisker. Multidomain or irregular precursors promote defect formation, plate-like morphologies, and scroll-like internal structures, whereas smooth, single-domain nanowhiskers favor the formation of symmetric, cylindrical nanotubes. These fundamental findings are graphically depicted in Figure 6. The presented conclusions explain the persistent structural diversity observed in WS_2_ nanotube synthesis and provide a mechanistic framework for controlling morphology through precursor engineering, enabling the rational design of structurally optimized nanotubes for advanced applications.

**Figure 6:**
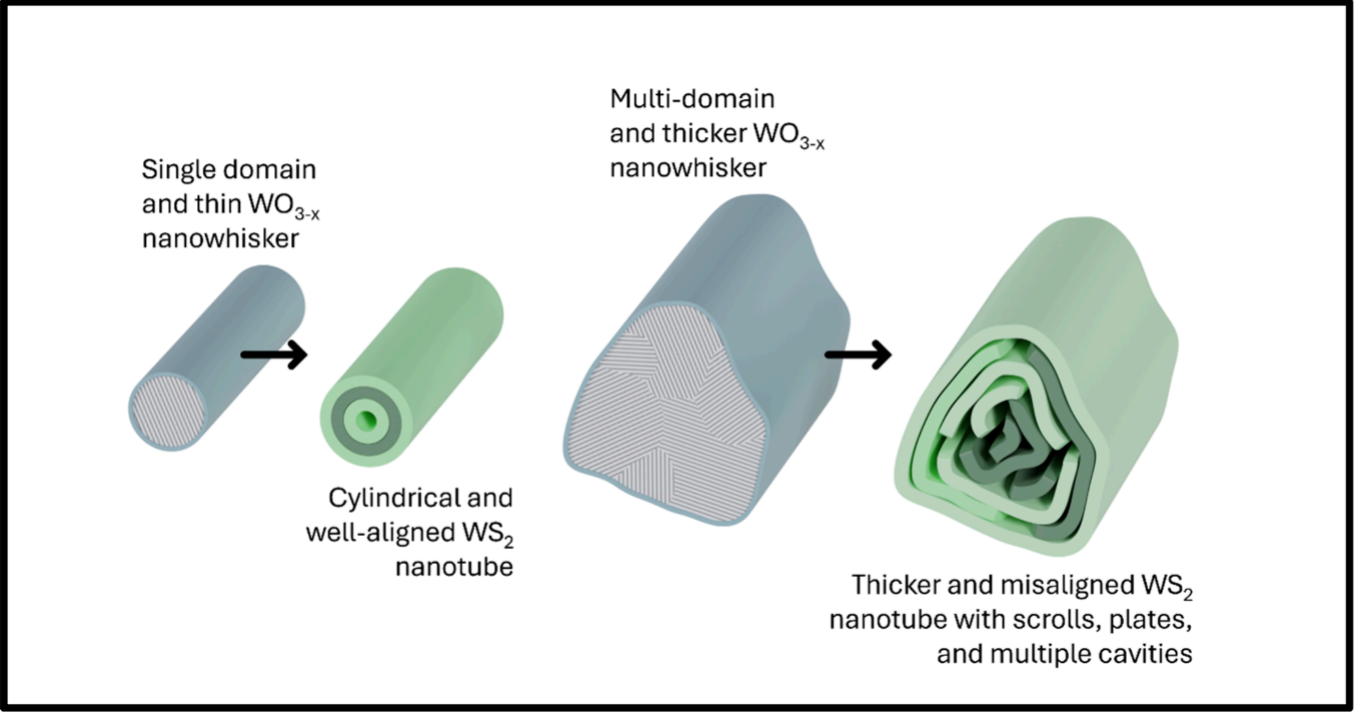
Graphical scheme of the structural relations of thin and single domain tungsten oxide nanowhisker precursors providing symmetrical and well-arranged WS_2_ nanotubes and thicker and multidomain nanowhiskers often transformed into thicker WS_2_ nanotubes with scroll-like structures, multiple cavities, and various other arrangements.

## Supplementary Material



## Notes

Additional experimental details, materials, and methods and detailed HRSTEM-HAADF and TEM images of cross sections and WS_2_ nanotubes, and SEM figures from in situ sulfidation of nanowhiskers (PDF)
