# Supplementary material for: Cross-Sectional Imaging of the WS2 Nanotube Formation Pathways
Source: Nano Lett. 2026 Apr 8;26(16):5566–71. doi: 10.1021/acs.nanolett.6c00855 (PMC13133909; doi:10.1021/acs.nanolett.6c00855)
Supplement: Supplementary file 1 [file nl6c00855_si_001.pdf]

Supplementary Information for:

Cross-Sectional Imaging of the WS_2 Nanotubes Formation Pathways

Kristyna Bukvisova,[a] Libor Novak,[a] Vojtech Kundrat[a,b]*
[a] Thermo Fisher Scientific, Vlastimila Pecha 12, 62700 Brno, Czech Republic
[b] Department of Chemistry, Faculty of Science, Masaryk University, Kamenice 5, Brno 62500, Czechia
*Corresponding author
Email: vojtechkundrat@mail.muni.cz
KEYWORDS: WS_2 nanotube, sulfidation, cross-section, lamella, electron microscopy, reaction mechanism

Experimental

Synthesis of precursor $W_{18}O_{49}$ nanowhiskers

A modified protocol[29] for the synthesis of W_5O_{14} ultralong nanowhiskers was used. The dark blue powder of tungsten hydrogen bronze ($H_{0.23\text{-}0.33}WO_3$, 1.0 g) was sealed in an evacuated quartz ampule (1×10^{-5} Pa) and submitted to a high temperature of 900 °C for 1 hour. The blue compact layer of ultralong nanowhiskers was obtained and further used.

Sulfidation in the μReactor and ex-situ cross-sectional TEM analysis

The procedure of sample preparation, handling, and operating the SEM with μReactor is described in detail in the previous study.[19] The standard modified procedure follows. The MEMS chip was gently scratched over the surface of the precursor nanowhisker layers. Ultralong nanowhiskers were deposited on its surface by this procedure. Followingly, the lamella was lifted out from the selected precursor $W_{18}O_{49}$ nanowhisker by the standard lift-out procedure (follows). Its cross-section TEM analysis was performed and signed as a measurement at 0 min. The MEMS chip with the rest of the nanowhisker was inserted into the μReactor, and the nanowhisker was sulfidated at 900 °C for 3 minutes in the mixture of H_2S and H_2 (100 and 50 Pa, respectively). Subsequently, another lamella was lifted out from the same nanowhisker, and the reaction continued again in the μReactor. In this manner, the sequences of TEM images in times 0, 3, 60 and 120 min were acquired.

Lift-out procedure, lamellae preparation

For the cross-sectional TEM lamella preparation, Thermo Fisher Helios 5 FX FIB-SEM was used. There were multiple extra steps needed to ensure that the nanowhisker or nascent nanotube was well-preserved in the final lamella. Carbon deposition covered the nanotube. The initial electron beam deposition was done using 2 kV and relatively low beam currents (< 500 pA) to minimize drift due to charging. The e-beam deposition was slow since the emission yield of secondary electrons needed to form the deposit is lower than from bulk substrates, in the areas close to the e-beam transparent windows. Therefore, it was useful to monitor the real protection layer thickness and proceed with the ion deposition when approx. 300 nm of continuous layer is deposited. For the same reason, the subsequent ion beam deposition was done at either lower kV (16 kV) or at 30 kV but with lower currents (approx. 100 pA). The trenches were milled as simple rectangle patterns, 1.5 μm wide and 7 μm deep (calibration for Si). Easylift insertion and positioning were inspected by the electron beam; the ion beam was used at a minimal current and only when the needle was very close to the lamella. After lift-out, standard procedure is used, and the final polishing is done at 5, 2, and 1 kV.

TEM analysis

Transmission electron microscopy was performed using Thermo Fisher Scientific Talos F200i in scanning (STEM) mode at an accelerating voltage of 200 kV and 35 pA probe current. Before imaging, the cross-sections were plasma cleaned gently.

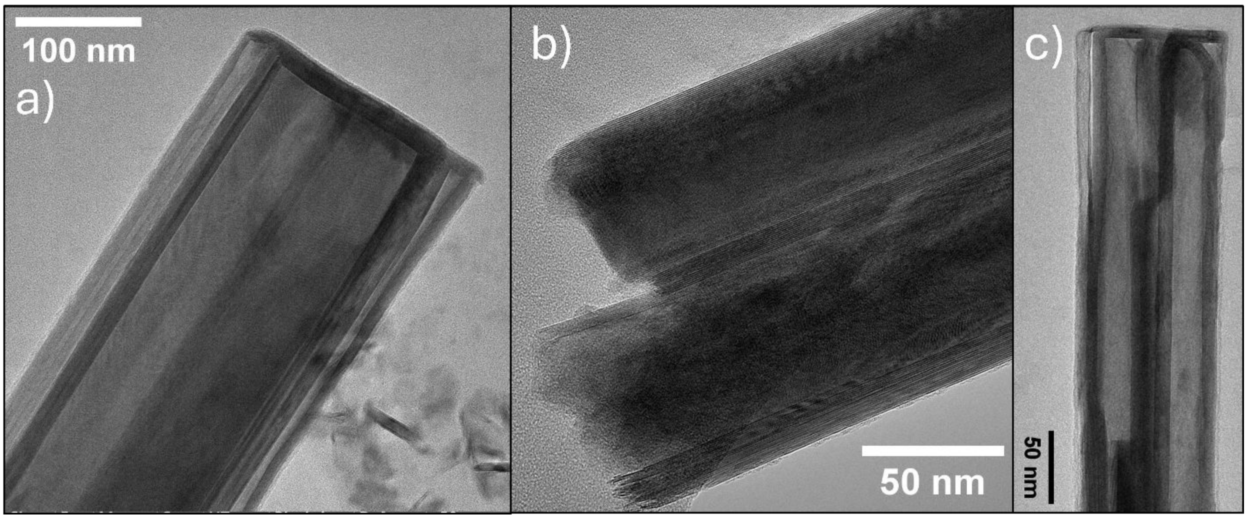

Figure S1. Various examples of imperfect thicker nanotubes with various defects.

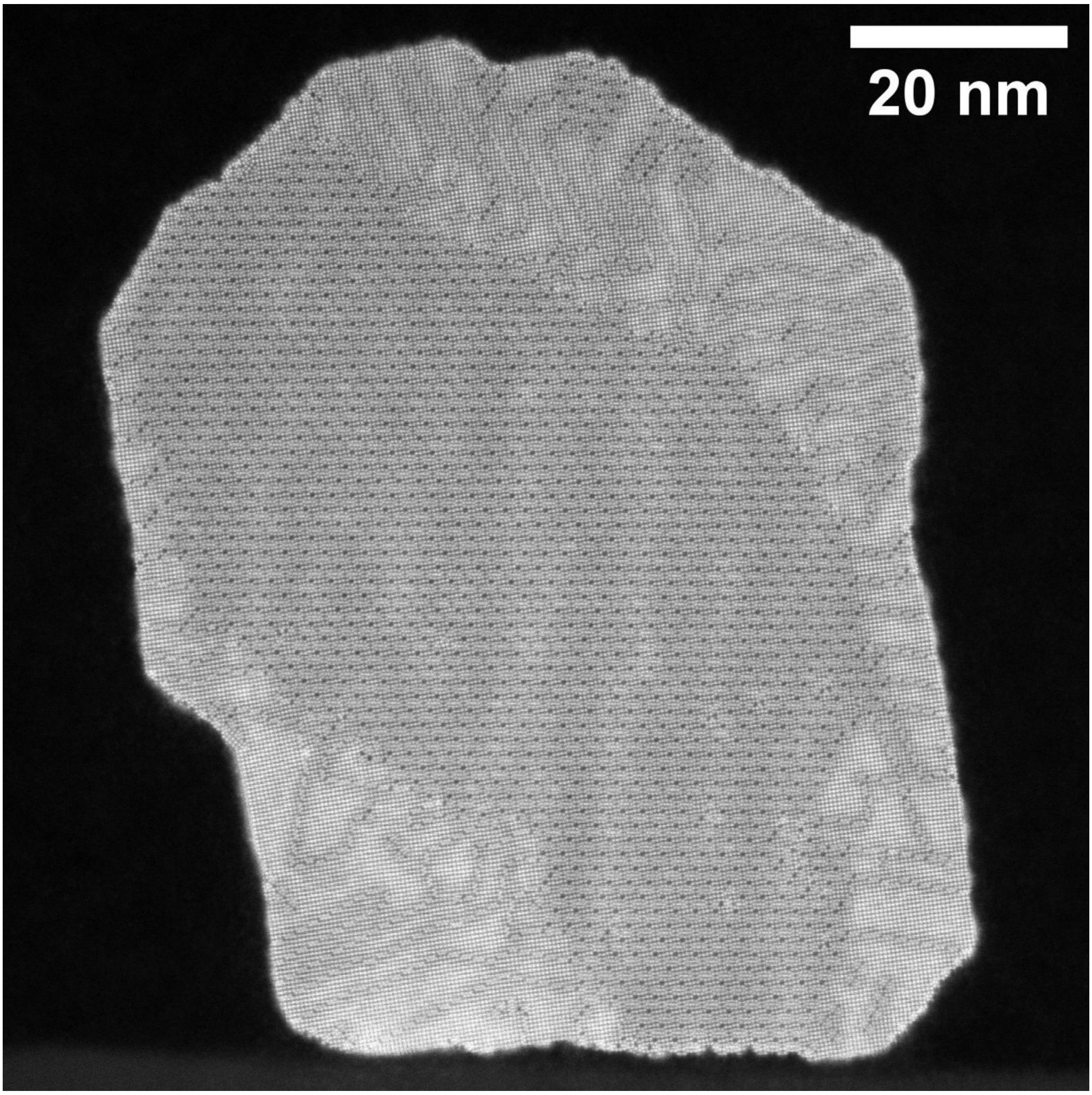

Figure S2. Detailed imaging of Figure 1c.

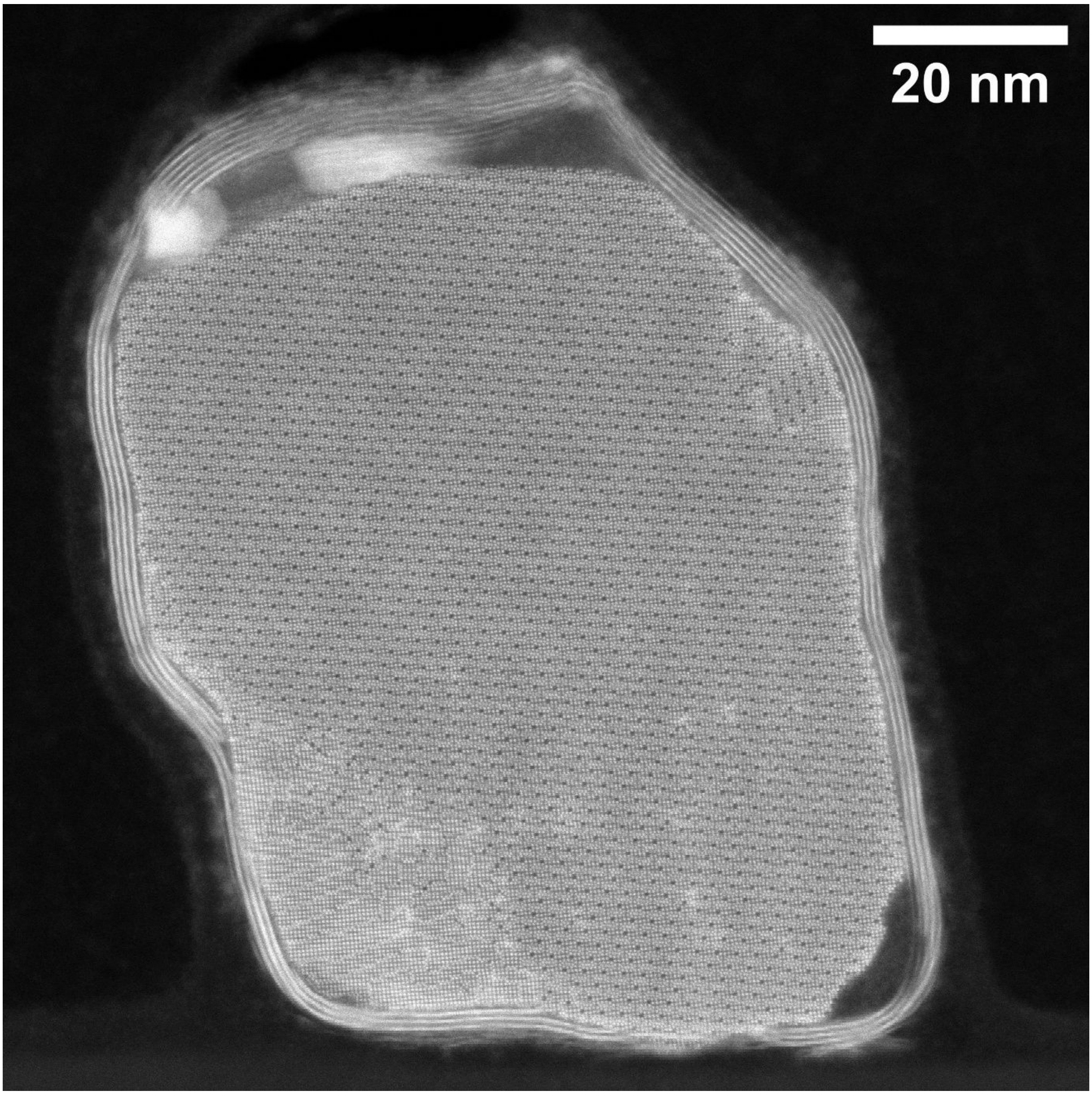

Figure S3. Detailed imaging of Figure 1d.

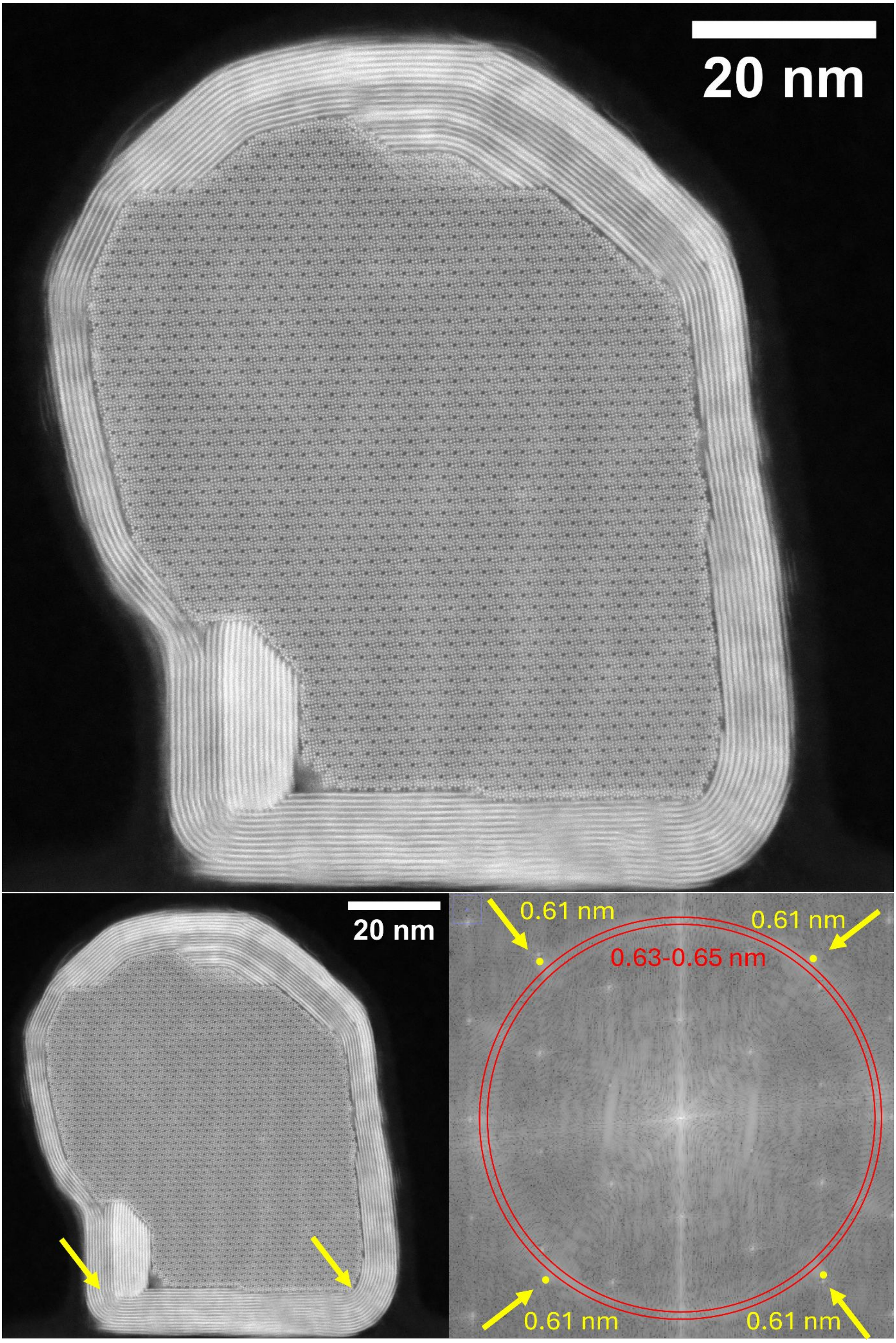

Figure S4. Detailed Figure 1e and FFT analysis showing the interlayer distance variations.

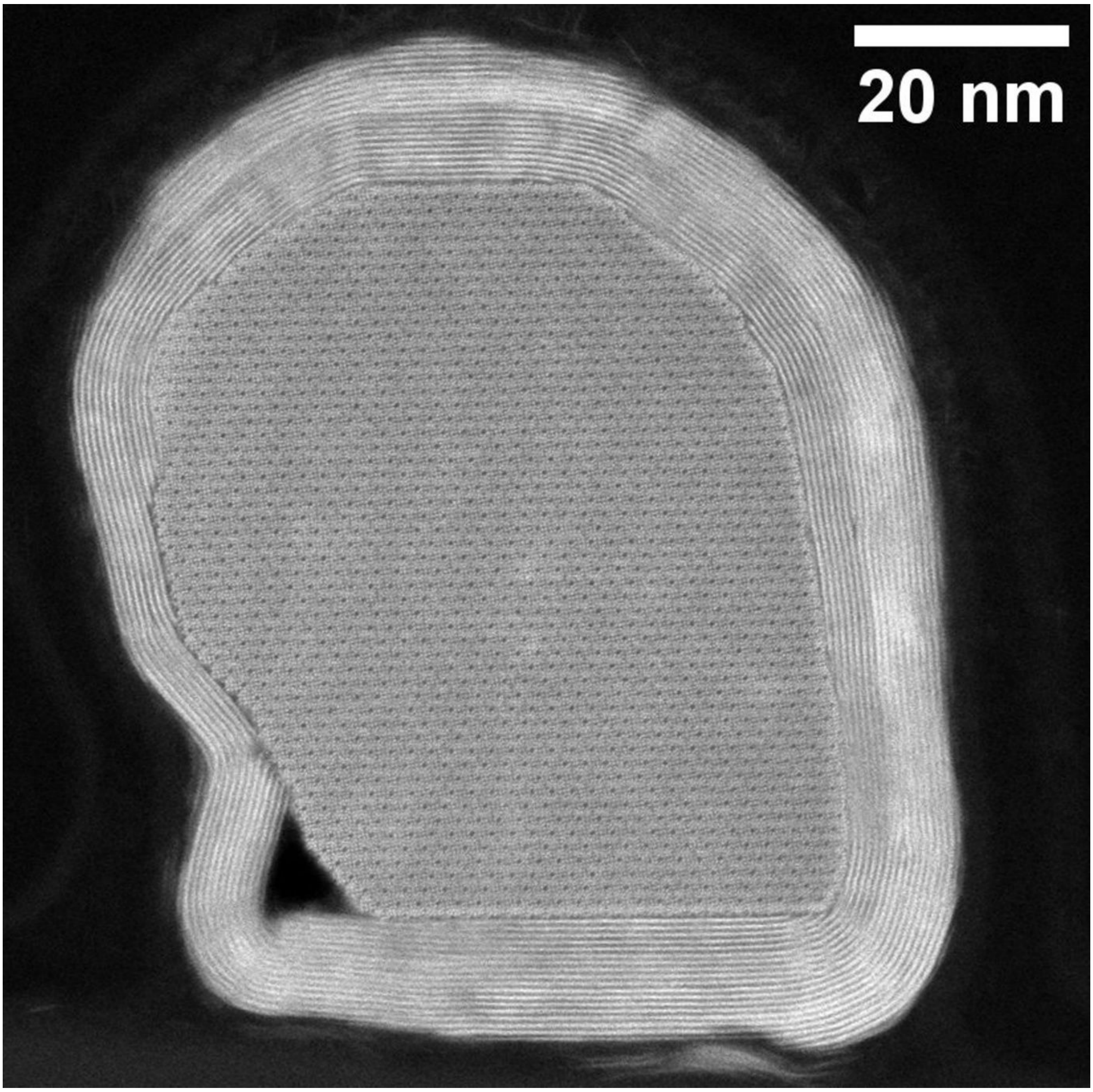

Figure S5. Detailed imaging of Figure 1f.

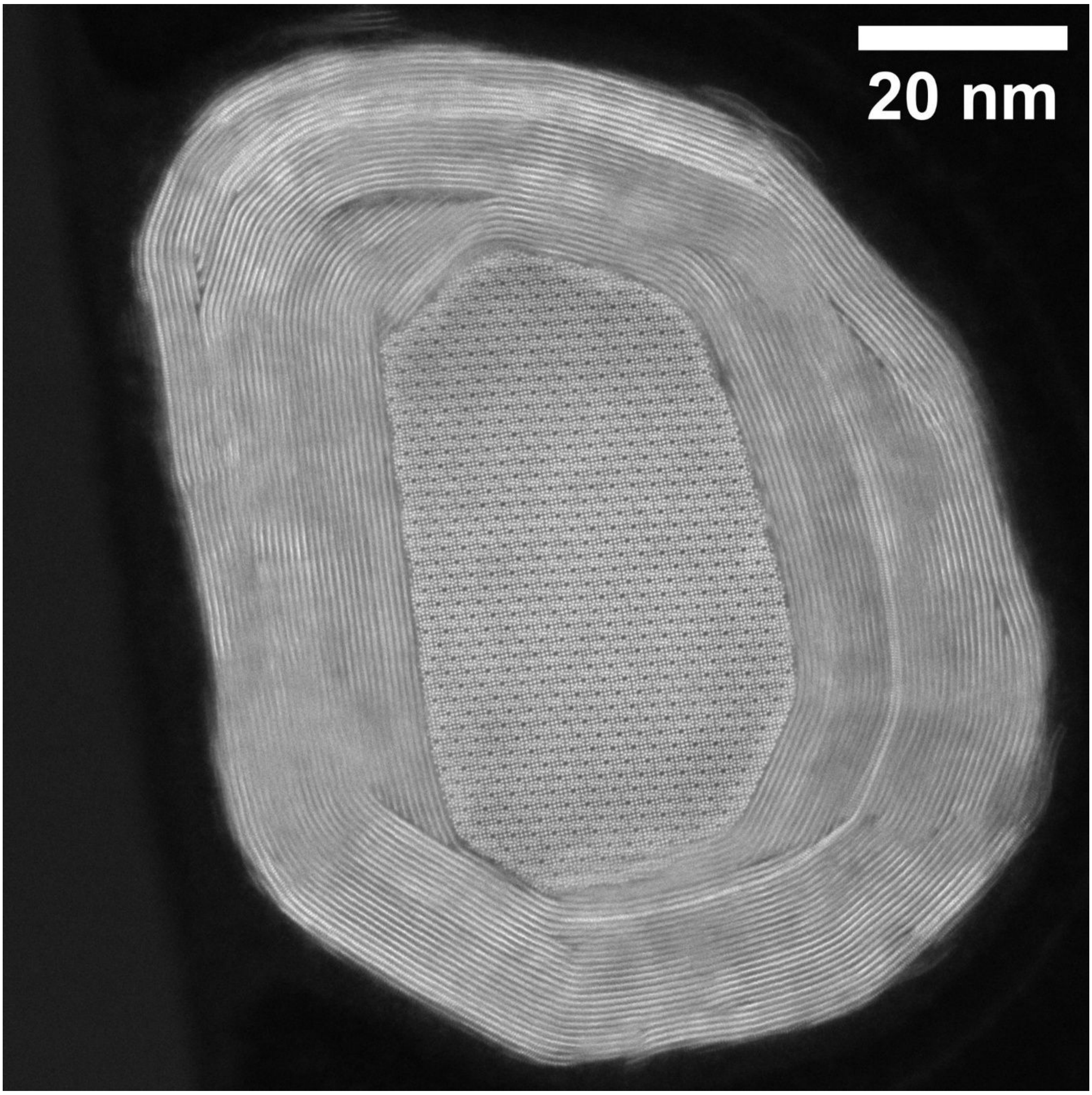

Figure S6. Detailed imaging of Figure 1g.

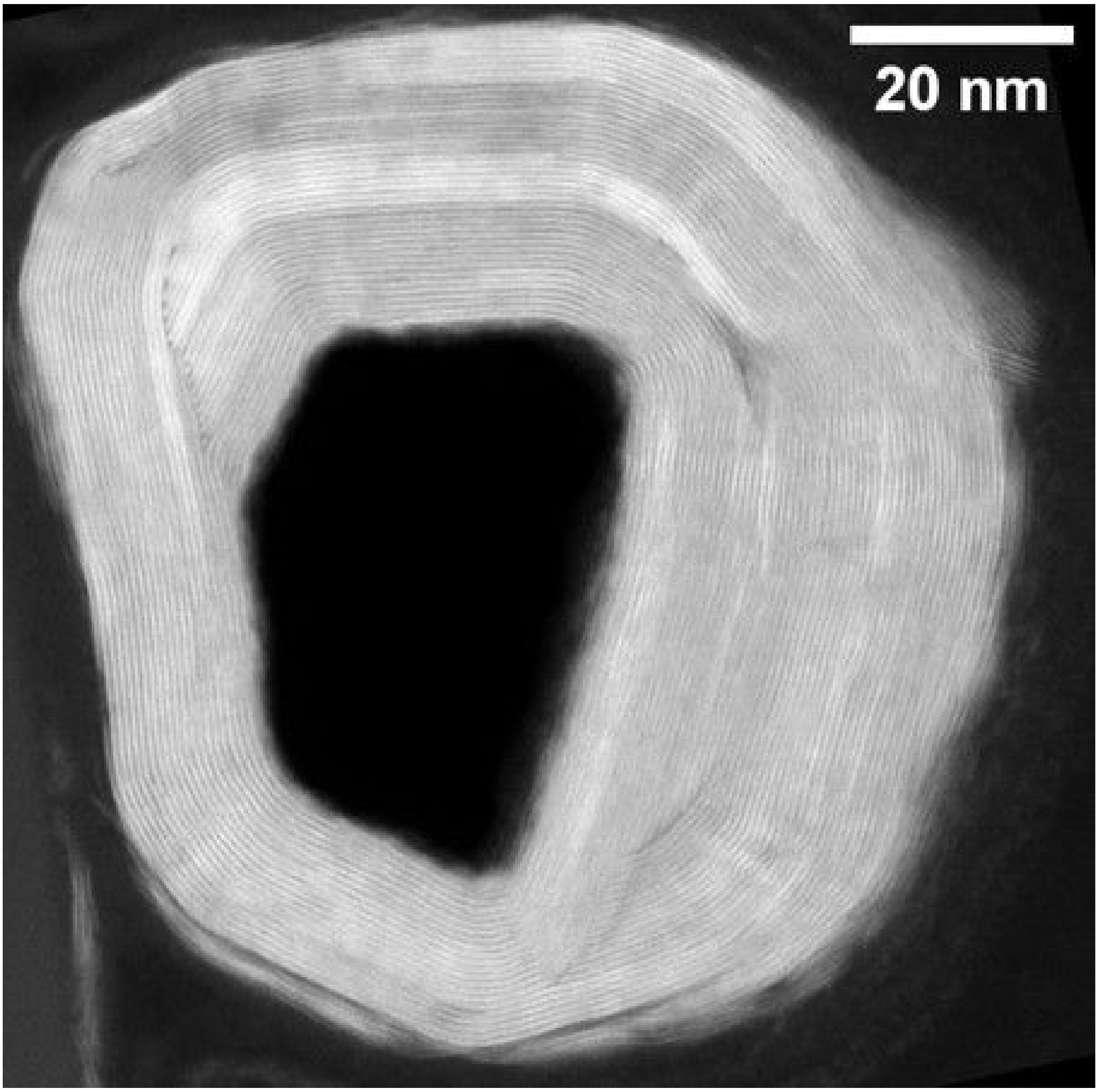

Figure S7. Detailed imaging of Figure 1h.

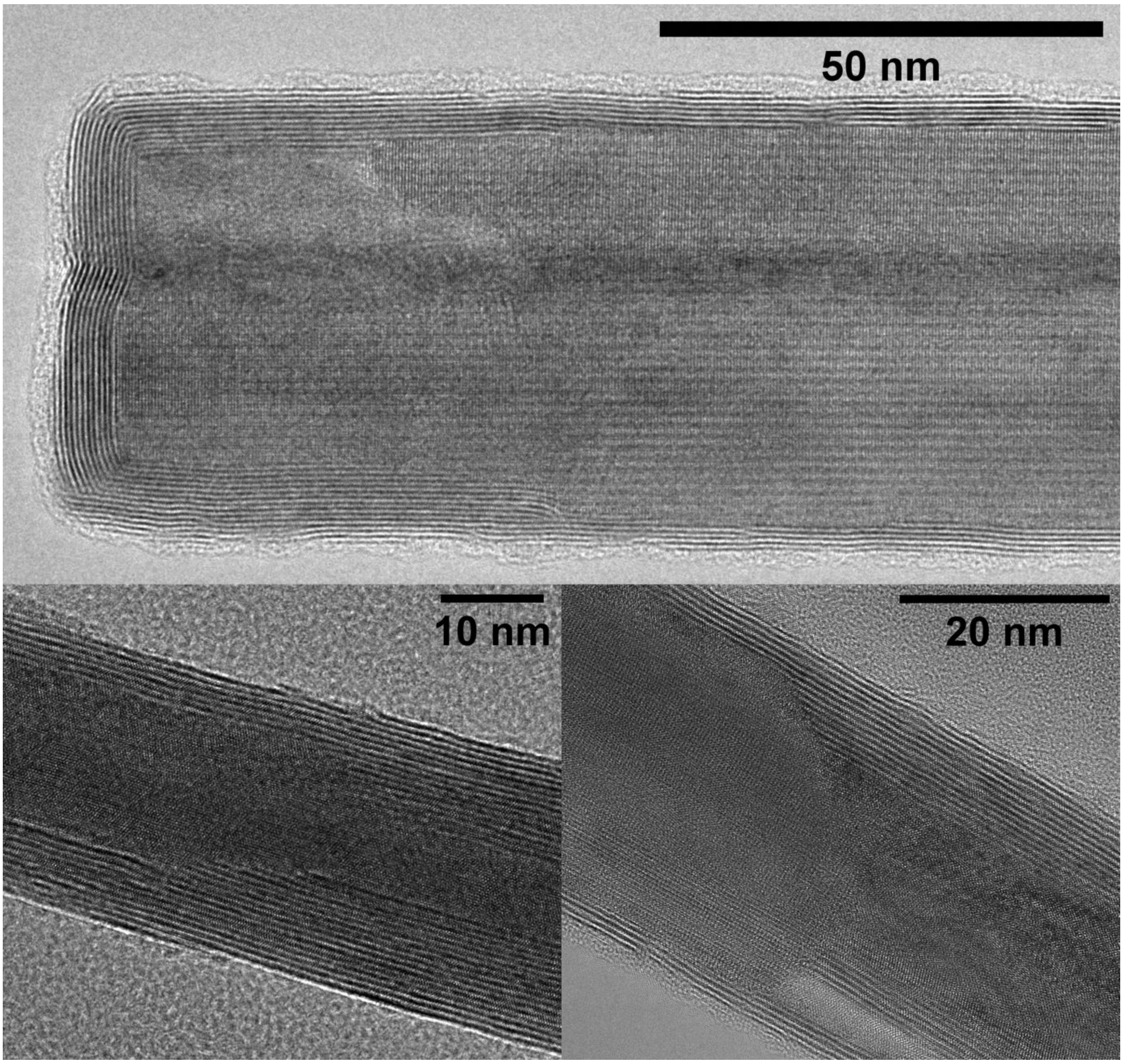

Figure S8. TEM analysis of three examples of nascent nanotubes focusing on the oxide-sulfide interface. New WS_2 layers form at the edge of the tungsten oxide core tip, indicating that the surface-inward growth mechanism is progressing along the nanotube rather than laterally.

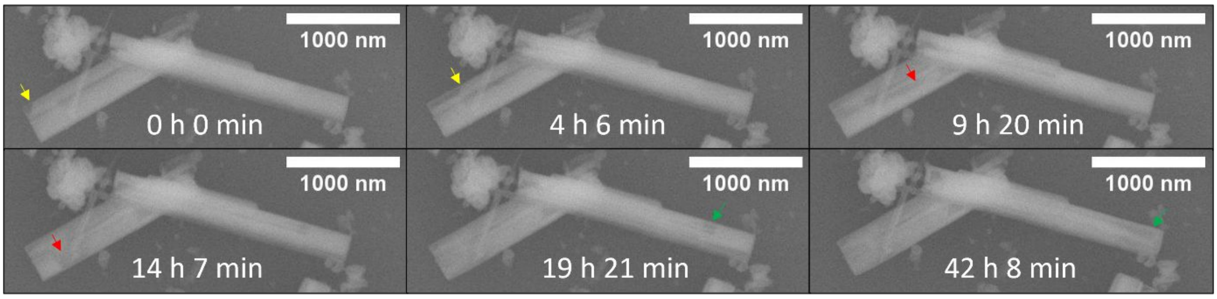

Figure S9. Formation of a thicker WS2 nanotube with multiple cavities and misaligned walls was observed as a sequence of SEM images in situ.

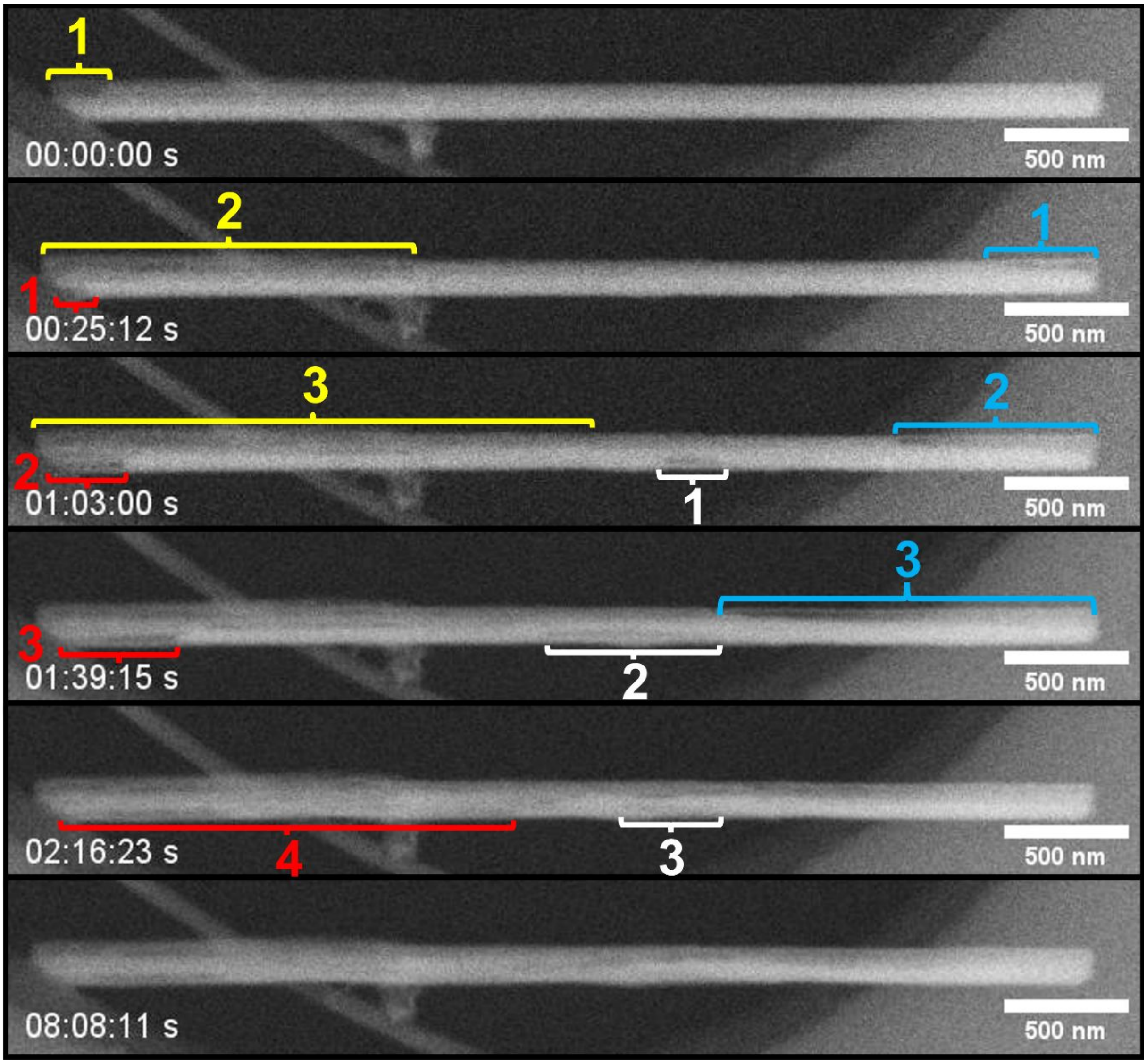

Figure S10. Detailed SEM sequence obtained in situ of sulfidation of multidomain WO_{3-x} nanowhisker. Various misaligned cavities are tracked by color brackets.
